# RO 90-7501 Enhances TLR3 and RLR Agonist Induced Antiviral Response

**DOI:** 10.1371/journal.pone.0042583

**Published:** 2012-10-03

**Authors:** Fang Guo, Jennifer Mead, Nishat Aliya, Lijuan Wang, Andrea Cuconati, Lai Wei, Kui Li, Timothy M. Block, Ju-Tao Guo, Jinhong Chang

**Affiliations:** 1 Department of Microbiology and Immunology, Drexel University College of Medicine, Doylestown, Pennsylvania, United States of America; 2 Institute for Hepatitis Virus Research, Hepatitis B Foundation, Doylestown, Pennsylvania, United States of America; 3 Institute of Hepatology, Peking University, Beijing, China; 4 Department of Microbiology, Immunology and Biochemistry, The University of Tennessee Health Science Center, Memphis, Tennessee, United States of America; University of Hong Kong, Hong Kong

## Abstract

Recognition of virus infection by innate pattern recognition receptors (PRRs), including membrane-associated toll-like receptors (TLR) and cytoplasmic RIG-I-like receptors (RLR), activates cascades of signal transduction pathways leading to production of type I interferons (IFN) and proinflammatory cytokines that orchestrate the elimination of the viruses. Although it has been demonstrated that PRR-mediated innate immunity plays an essential role in defending virus from infection, it also occasionally results in overwhelming production of proinflammatory cytokines that cause severe inflammation, blood vessel leakage and tissue damage. In our efforts to identify small molecules that selectively enhance PRR-mediated antiviral, but not the detrimental inflammatory response, we discovered a compound, RO 90–7501 (‘2’-(4-Aminophenyl)-[2,5′-bi-1H-benzimidazol]-5-amine), that significantly promoted both TLR3 and RLR ligand-induced IFN-β gene expression and antiviral response, most likely *via* selective activation of p38 mitogen-activated protein kinase (MAPK) pathway. Our results thus imply that pharmacological modulation of PRR signal transduction pathways in favor of the induction of a beneficial antiviral response can be a novel therapeutic strategy.

## Introduction

Infection of viruses is promptly recognized by host innate pattern recognition receptors (PRRs), including Toll-like receptors (TLRs), RIG-I-like receptors (RLRs), NOD-like receptors and C-type lectins [1], [2]. This results in production of type I interferons (IFN), proinflammatory cytokines and chemokines that orchestrate the elimination of the pathogens. The essential role of the PRR-mediated innate immune response in defending against microorganism infection has been extensively demonstrated *in vivo* in murine models with knockout of the genes encoding either specific PRRs or their key signaling components (reviewed in [3], [4]). However, like adaptive immunity, the innate immune response can also be detrimental to hosts. Indeed, in many occasions, it is not the viral replication itself, but the overwhelming production of proinflammatory cytokines that causes severe inflammation, tissue damage, blood vessel leakage and occasionally permeabilization of the blood brain barrier that leads to the penetration and infection of central nervous system by viruses [5], [6], [7]. In addition, because of the vital role of PRRs in defending against virus infection, pharmacological activation of PRR-mediated innate host response has been extensively explored as a broad-spectrum antiviral approach [8], [9], [10]. However, systematic administration of the PRR agonists in doses necessary to achieve antiviral effects is usually associated with significant adverse reactions, due to the activation of a wide-spectrum of cellular responses and massive production of proinflammatory cytokines [11], [12], [13], [14].

TLRs and RLRs are two major types of PRRs that recognize virus infection and induce innate immune response. Interestingly, induction of type I IFNs, the primary antiviral cytokines, and other proinflammatory cytokines upon activation of TLRs and RLRs is controlled by multiple overlapping, but distinct signal transduction pathways (reviewed in [15]). While activation of nuclear factor kappa-light-chain-enhancer of activated B cells (NFκB) and distinct mitogen-activated protein kinase (MAPK) pathways are critical for the production of many proinflammatory cytokines and chemokines, activation of the interferon regulatory factor 3 (IRF3) (or IRF7) pathway is only required for induction of type I IFNs as well as a group of antiviral proteins, such as IFIT1, guanylate binding protein 1 and zinc finger antiviral protein [16], [17], [18]. In addition, although the three MAP kinases, p38, ERK and JNK, can be activated by TLR and RLR agonists and viral infection [19], [20], each of the three MAPKs has been demonstrated to play distinct roles in regulating the expression of type I IFN and other proinflammatory genes [21], [22], [23]. For example, it has been shown recently that ERK activation is required for TLR3-induced chemokine production in murine dendritic cells, whereas JNK activation has a negative regulatory effect on chemokine production [24]. It is, therefore, possible to pharmacologically modulate the virus- and/or PRR-agonist-induced innate immune response by targeting distinct signal transduction pathways to selectively enhance the antiviral response, but alleviate the detrimental inflammatory response. It is conceivable that such a therapy should be effective to a broad spectrum of virus infections, either alone or in combination with PRR agonists.

In order to discover compounds with the expected pharmacological property, we set out to establish reporter cell lines for high throughput screening of small molecules that selectively enhance TLR3 ligand-induced IFN-β gene expression, but do not affect NFκB activation, which is a central player in the induction of proinflammatory cytokines, but plays a less prominent role in type I IFN gene expression [25]. Our initial high throughput screening campaign has thus far identified a compound, RO 90–7501, that selectively enhances TLR3 and RLR ligand-induced IFN-β gene expression and antiviral response, most likely *via* activation of the p38 MAPK pathway, but not the NFκB or IRF3 pathway. Our results have thus proved the concept that pharmacological modulation of TLR3 agonist-induced innate immune response can be a viable antiviral therapeutic strategy.

## Results

### Establishment and characterization of reporter cell lines

**Figure 1:**
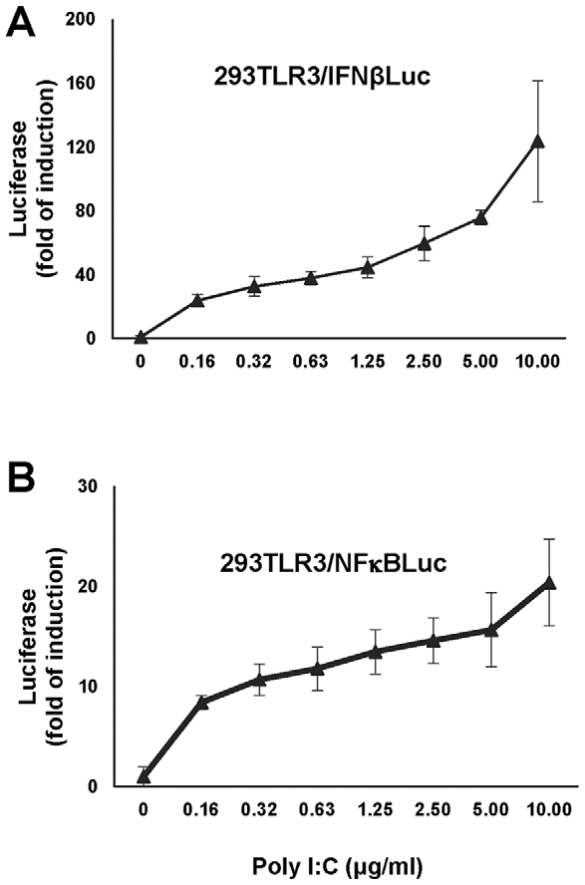
Establishment of 293TLR3HA-derived IFN-β promoter and NFκB reporter cell lines. To test the dose dependent response of 293TLR3/IFNβLuc (A) and 293TLR3/NFκBLuc (B) cells to TLR3 ligand, cells were mock treated or treated with the indicated concentrations of poly I:C for 16 h. Promoter activities were expressed as fold of induction of luciferase activity compared to mock treated control (mean +/− standard derivations, n = 3).

**Figure 2:**
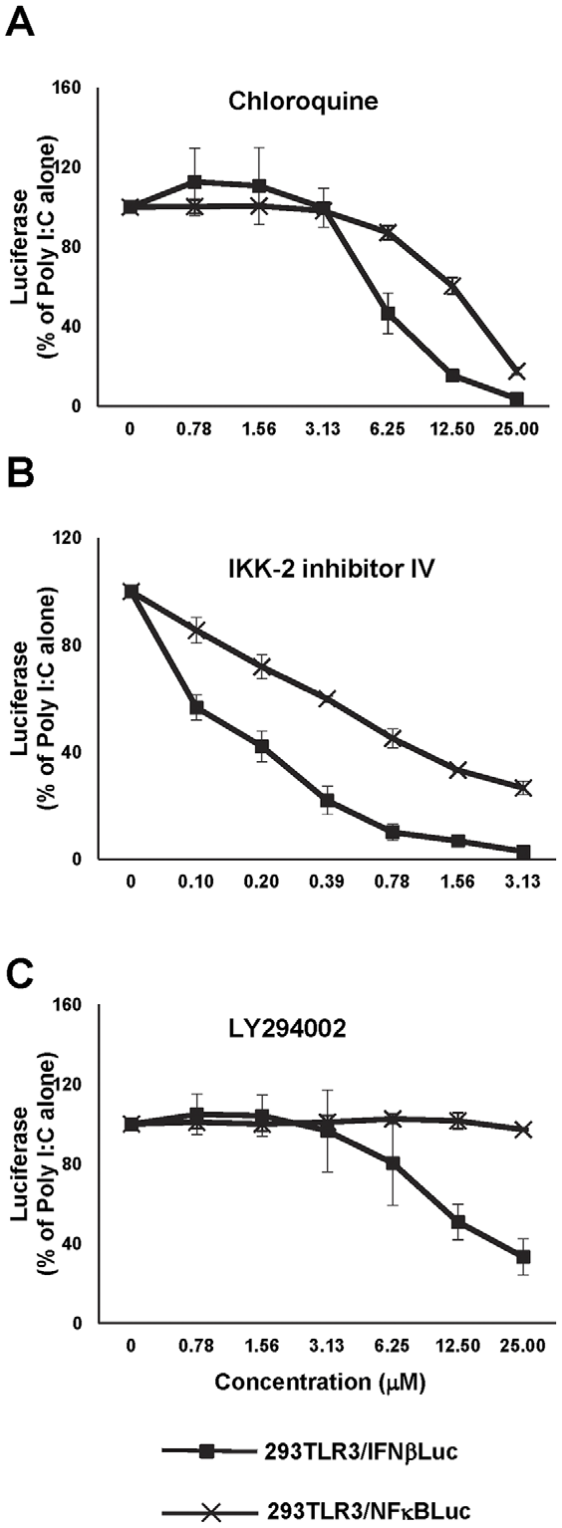
Characterization of 293TLR3HA-derived IFN-β promoter and NFκB reporter cell lines. 293TLR3/IFNβLuc and 293TLR3/NFκBLuc cells were treated with 2 µg/ml of poly I:C in the absence or presence of the indicated concentrations of compounds for 16 h. (A) Endosome acidification inhibitor chloroquin. (B) NFκB pathway IKK-2 inhibitor. (C) PI3k inhibitor LY294002. Luciferase activities were determined and the effects of the tested compounds on the promoter activity were normalized and expressed as a percentage of luciferase activity obtained from the cells treated with poly I:C alone (mean +/− standard derivations, n = 3).

Type I IFNs are the primary antiviral cytokines induced by the PRR agonist-activated innate immune response, whereas NFκB is the key transcription factor for the induction of proinflammatory cytokines and chemokines, but plays a less important role in IFN-β gene activation [25]. First, we set out to establish cell-based assays suitable for high throughput screening of compounds that selectively enhance TLR3 agonist-induced type I IFN production, without enhancing the activation of NFκB. Two TLR3-expressing HEK293 (293TLR3HA, Invivogen)-derived stable reporter cell lines that express firefly luciferase under the control of a human IFN-β promoter [26] (designated 293TLR3/IFNβLuc) or an artificial promoter containing consensus NFκB binding sites (Clontech) (designated 293TLR3/NFκBLuc) were established. As shown in Fig. 1, poly I:C treatment of both the reporter cell lines induced luciferase expression in a concentration dependent manner. As expected, poly I:C treatment of 293/IFNβLuc cell line without TLR3 fails to induce luciferase expression (data not shown). Moreover, as shown in Figs. 2A and B, while the poly I:C-induced luciferase expression was dose-dependently inhibited by chloroquine (a lysosome acidification inhibitor) and IKK-2 inhibitor IV (an IKK-β inhibitor) in both 293TLR3/IFNβLuc and 293TLR3/NFκBLuc cells, LY294002, an inhibitor of phosphoatidylinositol 3-kinase (PI3K) that is required for activation of IRF3 [27], only inhibited poly I:C-induced luciferase expression in 293TLR3/IFNβLuc, but not in 293TLR3/NFκBLuc cells (Fig. 2C). These results thus validated that the luciferase expression in 293TLR3/IFNβLuc and 293TLR3/NFkBLuc cells was controlled by the functional IFN-β promoter and the NFκB responsive element, respectively.

### RO 90–7501 selectively enhances IFN-β, but not NFκB activation

**Figure 3:**
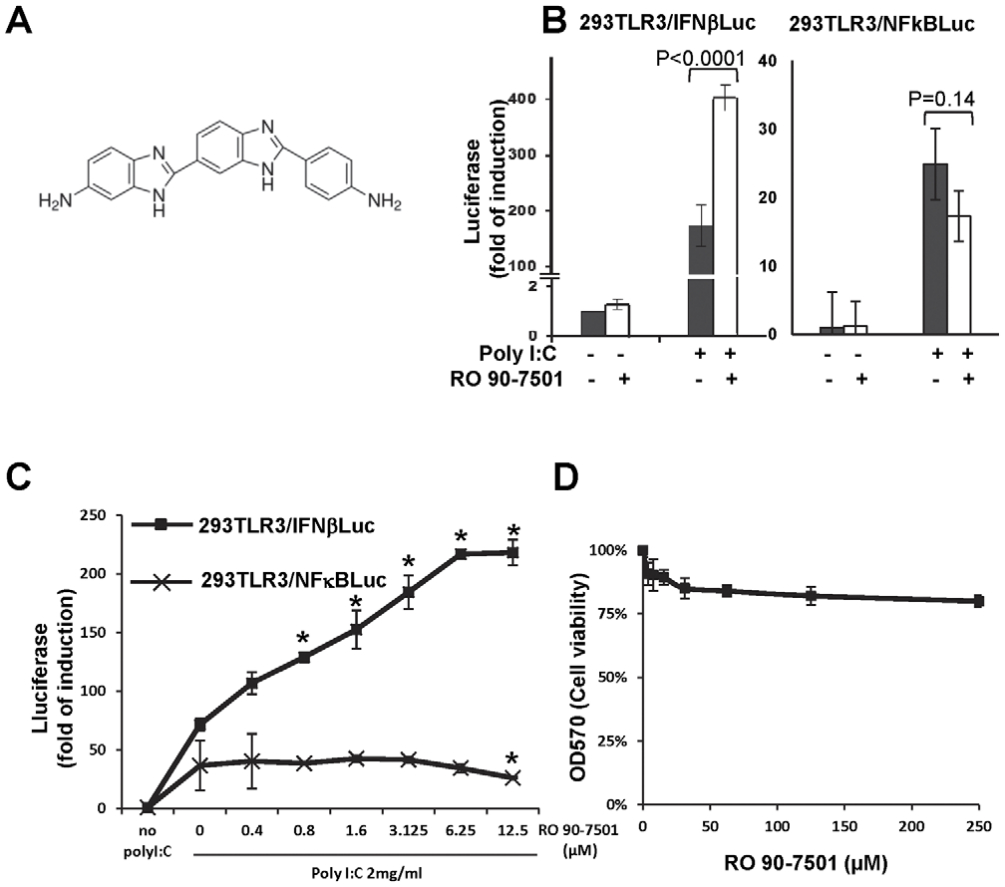
RO 90–7501 selectively enhances the activation of IFN-β promoter but not NFκB reporter by poly I:C. (A) Structure of RO 90–7501. (B) 293TLR3/IFNβLuc and 293TLR3/NFκBLuc cells were mock treated or treated with 10 µM of RO 90–7501, 2 µg/ml of poly I:C, alone or in combination for 16 h. Luciferase activities were normalized to mock-treated controls and expressed as fold of induction (mean +/− standard deviation, n = 8). P values were calculated using the two-tailed student t test. (C) 293TLR3/IFNβLuc and 293TLR3/NFκBLuc cells were mock treated or treated with 2 µg/ml of poly I:C in the absence or presence of the indicated concentrations of RO 90–7501 for 16 h. The effects of RO 90–7501 on the IFN-β promoter and NFκB reporter activation were normalized to mock-treated controls and expressed as fold of induction of luciferase activity (mean +/− standard derivation, n = 3). P values were calculated using the two-tailed student t test, with * indicates P<0.05 compared to poly I:C treated alone. (D) 293TLR3/IFNβLuc cells were mock treated or treated with indicated concentrations of RO 90–7501 for 16 h. Cell viability was determined by a MTT assay and the results were plotted as the dose-dependent absorbance values at 570 nm.

Through screening of 1,280 small molecules in the library of pharmacologically active compounds (LOPAC) obtained from Sigma, we were able to identify a compound, RO 90–7501 (‘2’-(4-Aminophenyl)-[2,5′-bi-1H-benzimidazol]-5-amine) (Fig. 3A), that itself affected neither IFN-β nor NFκB promoter activity, but significantly enhanced poly I:C-induced IFN-β promoter activation and inhibited the activation of NFκB in a dose-dependent manner (Figs. 3B and C). While its enhancement of IFN-β promoter activation was statistically significant at doses ranging from 0.8 to 12.5 µM, its inhibition on NFκB activation was only statistically significant at the highest dose tested. No cytotoxicity was observed at concentrations up to 250 µM of RO 90–7501 by a MTT assay (Fig. 3D).

### RO 90–7501 enhances poly I:C-induced activation of IFN-β promoter, but does not change the activation kinetics

**Figure 4:**
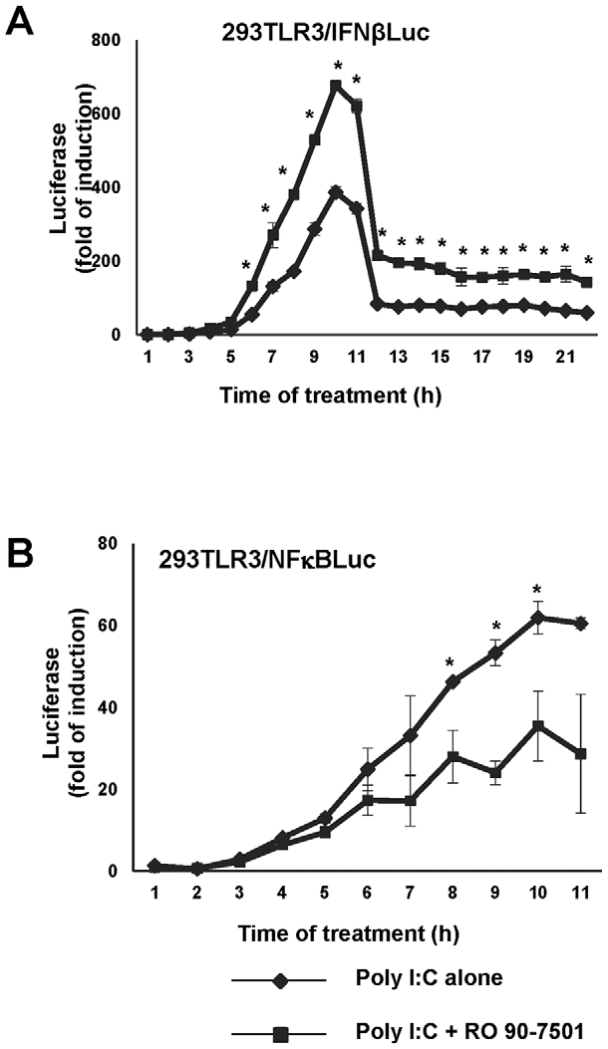
RO 90–7501 does not alter the kinetics of IFN-β promoter and NFκB activation by poly I:C. 293TLR3/IFNβLuc and 293TLR3/NFκBLuc cells were treated with 2 µg/ml of poly I:C alone or in the presence of 10 µM of RO 90–7501. At the indicated time after treatment, the cells were lysed and luciferase activity was measured and expressed as fold of induction over untreated controls (mean +/− standard derivations, n = 3). P values were calculated using the two-tailed student t test, with * indicates P<0.05 compared to poly I:C treated alone.

The observed increase of IFN-β promoter activity caused by RO 90–7501 may be due to the enhancement of poly I:C activation, or alternatively due to the disruption of a feedback inhibition pathway. In order to distinguish these two possibilities, a time-course study was performed. As shown in Fig. 4A, the presence of RO 90–7501 increased the peak level of IFN-β promoter activity at 10 h of poly I:C treatment, but the promoter activity declined with a similar kinetics since the peak time in the absence or presence of RO 90–7501. Consistent with results shown in Figs. 2B and C, RO 90–7501 inhibited the activation of NFκB (Fig. 4B). The results thus imply that RO 90–7501 most likely targets a molecular event in the activation, but not negative feedback regulation of TLR3 signal transduction pathway to prolong the activation of IFN-β promoter.

### RO 90–7501 modulates TLR3 signal transduction, but has no effect on cells that TLR3 signal transduction is not activated

**Figure 5:**
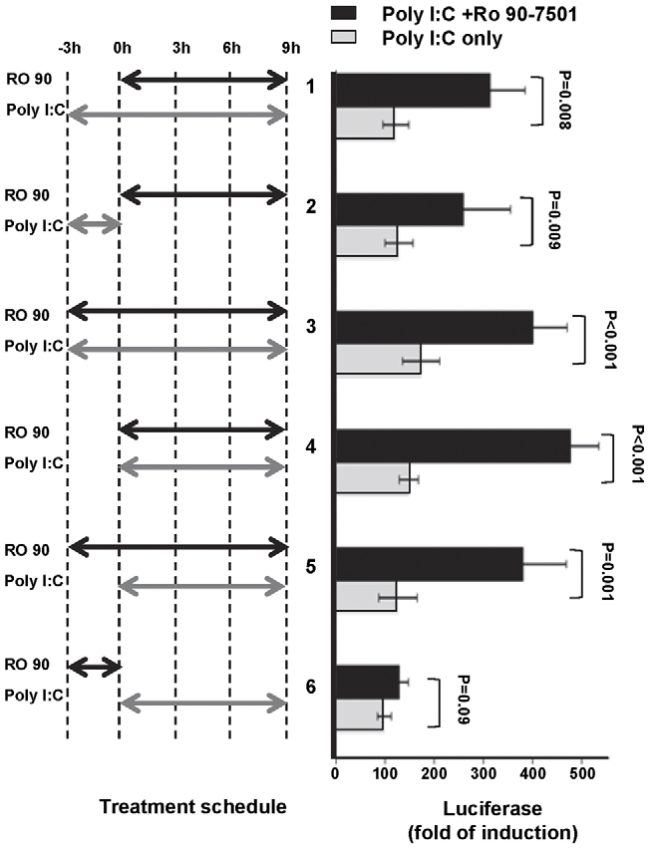
Time-of-addition experiment. 293TLR3/IFNβLuc cells were treated either with 2 µg/ml poly I:C alone or in combination with 10 µM of RO 90–7501 by the six different treatment schedules depicted on the left panel. Luciferase activities in the cell lysate were measured and expressed as fold of induction over poly I:C untreated controls (mean +/− standard derivations, n = 3). P values were calculated using the two-tailed student t test.

The differential regulation of RO 90–7501 on the activation of IFN-β promoter and NFκB induced by poly I:C suggests that the compound modulates TLR3 signal transduction, but does not target ligand RNA or TLR3 itself. In agreement with this notion, we observed in a time-of-addition experiment that RO 90–7501 was able to efficiently enhance poly I:C activation of IFN-β promoter when added 3 h before, at the same time or even 3 h after the addition of poly I:C into the culture media (Fig. 5). However, the compound did not enhance poly I:C-induced IFN-β promoter activation when the cells were pretreated with the compound for 3 h, followed by treatment with poly I:C in the absence of RO 90–7501 for an additional 9 h (treatment schedule 6). These results indicate that RO 90–7501 has no effect on IFN-β promoter activation in the absence of TLR3 ligand, suggesting the compound should be pharmacologically active only in virally-infected, but not un-infected cells.

### R0-90 enhances endogenous IFN-β gene expression in both 293TLR3HA and THP-1 cells

**Figure 6:**
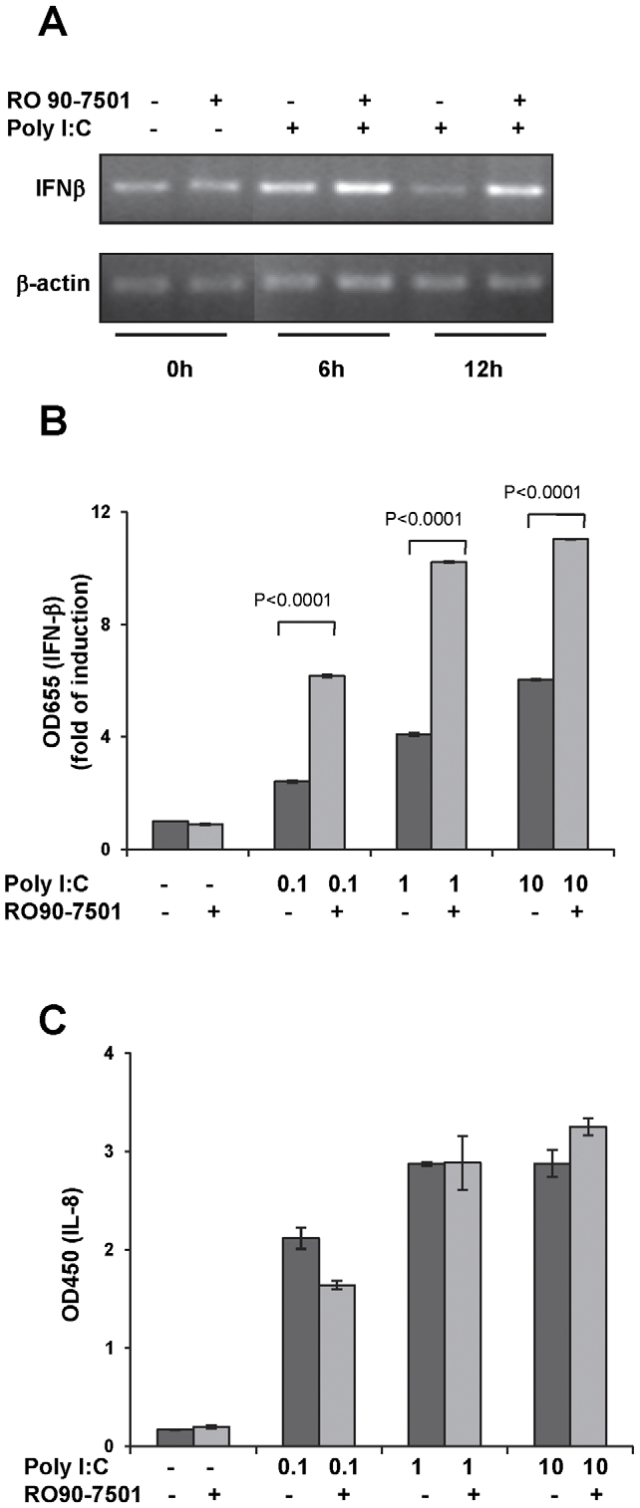
RO 90–7501 enhances the expression of endogenous IFN-β gene in both 293TLR3HA and human macrophage cell line THP-1. (A) 293TLR3HA cells were mock treated or treated with 10 µM of RO 90–7501, 2 µg/ml poly I:C, alone or in combination. The cells were harvested at the indicated time since start of the treatment. The levels of IFN-β and β-actin mRNAs were determined by semi-quantitative RT-PCR. (B) THP-1 cells were mock treated or treated with the indicated concentrations of poly I:C in the absence or presence of 10 µM RO 90–7501 for 6 h. The levels of secreted type I IFN were then determined with HEK-Blue IFN-α/β cells using culture media transferred from treated THP-1 cells. The level of ISG54 promoter driven SEAP expression in the supernatant was then determined with QUANTI-Blue and expressed as absorbance values at 655 nm, normalized to mock treated controls (mean +/− standard derivations, n = 8). P values were calculated using the two-tailed student t test. (C) 293TLR3HA cells were mock treated or treated with the indicated concentrations of poly I:C in the absence or presence of 10 µM RO 90–7501 for 6 h. Secreted IL-8 in culture media from treated 293TLR3HA cells were determined with a human IL-8 ELISA kit, and expressed as absorbance values at 450 nm (mean +/− standard derivations, n = 3).

To further confirm the observations made with the reporter cell lines, we measured the effects of RO 90–7501 on the TLR3 ligand-induced endogenous IFN-β and IL-8 (a chemokine) expression in 293TLR3HA and THP-1 (a human monocytic cell line) cells. Consistent with the results obtained with reporter assays, while treatment of the cells with RO 90–7501 alone failed to induce IFN-β gene expression, the compounds significantly increased the induction of IFN-βmRNA in the presence of poly I:C in 293TLR3HA cells (Fig. 6A), and the secretion of type-I IFN in THP-1 cells (Fig. 6B), but not IL-8 secretion (Fig. 6C).

### RO 90–7501 enhances IFN-β promoter activation by a cytoplasmic RLR agonist

**Figure 7:**
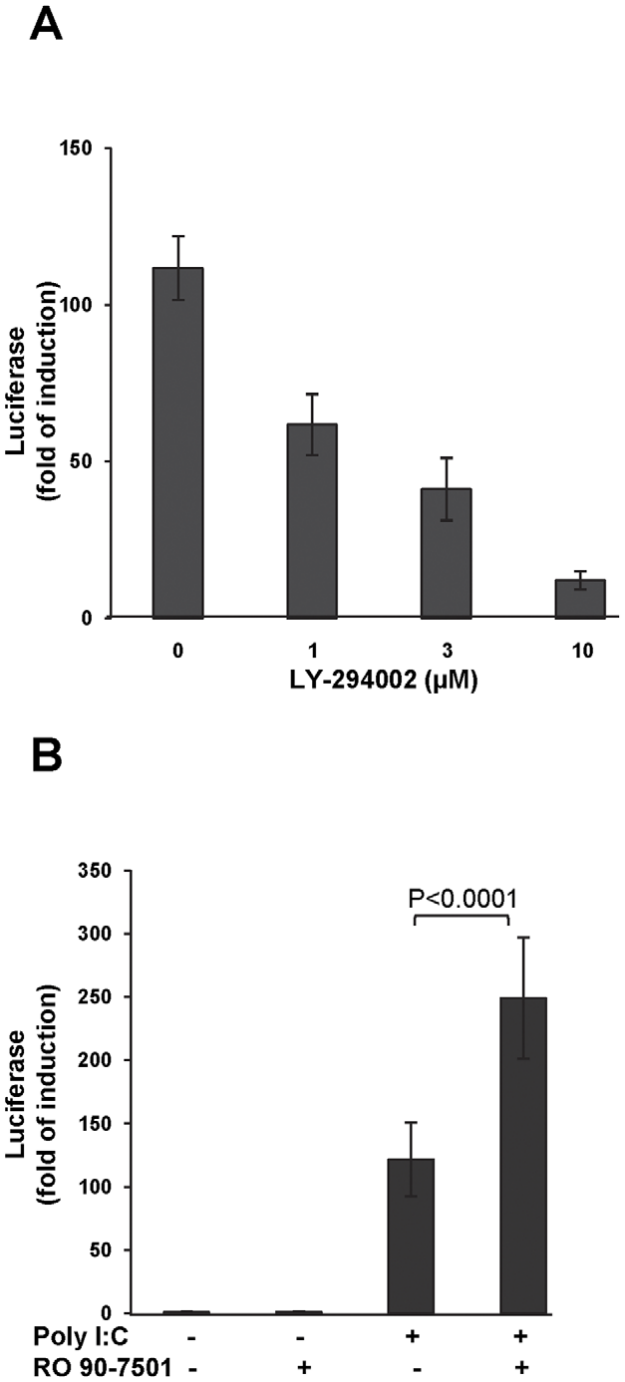
RO 90–7501 enhances the cytoplasmic RLR ligand-induced IFN-β expression. (A) 293/IFNβLuc cells, which are deficient of TLR3, were transfected with poly I:C and mock treated or treated with the indicated concentrations of PI3K inhibitor LY294002 for 16 h. Luciferase activities in the cell lysate were measured and expressed as fold of induction over mock-transfected controls (mean +/− standard derivations, n = 24). (B) 293/IFNβLuc cells were either mock transfected (no RNA) or transfected with poly I:C. The cells were then mock treated or treated with 10 µM of RO 90–7501 for 16 h. Luciferase activities in the cell lysate were measured and expressed as fold of induction over mock-transfected controls (mean +/− standard derivations, n = 32). P values were calculated using the two-tailed student t test.

Because the downstream signaling pathways leading to activation of IFN-β and inflammatory cytokine gene expression are largely shared by both TLR3 and cytoplasmic RLRs, we set out to test whether RO 90–7501 could also enhance the activation of the IFN-β promoter elicited by cytoplasmic delivery of double stranded RNA, which activates both RIG-I and MDA5 [26], [28], into 293/IFNβLuc cells that do not express TLR3. As shown in Fig. 7, while LY294002, an inhibitor of PI3K that is essential for activation of IRF3 by RIG-I/MDA5 [29], dose-dependently inhibited IFN-β promoter-driven luciferase expression (Fig. 7A), RO 90–7501 significantly enhanced the IFN-β promoter activation by cytoplasmic-delivered poly I:C (Fig. 7B). Hence, our results imply that RO 90–7501 most likely targets a shared IFN-β induction pathway activated by both TLR3 and RLRs.

### RO 90–7501 enhances the antiviral response induced by poly I:C

**Figure 8:**
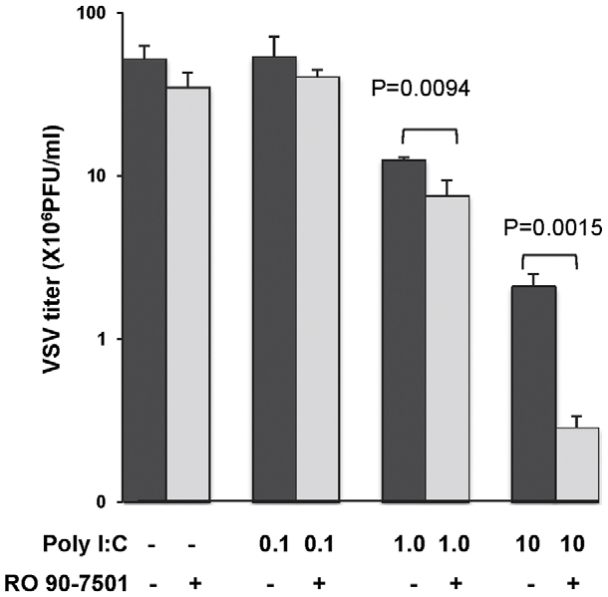
RO 90–7501 enhances poly I:C-induced antiviral activity. 293TLR3HA cells were left untreated or treated with indicated concentrations of poly I:C in the absence or presence of 10 µM RO 90–7501 for 12 h. Cells were then infected with VSV at a MOI of 0.001. Culture media were harvested 16 h post infection and the virus yields were determined by a plaque assay and presented as number of plaque forming units (pfu) per milliliter medium (mean +/− standard derivations, n = 3). P values were calculated using the student t test.

To investigate whether the enhanced IFN-β response by RO 90–7501 could result in an enhanced antiviral response, 293TLR3HA cells were mock treated, or treated with poly I:C, RO 90–7501, alone or in combination for 12 h. cells were then challenged with vesicular stomatitis virus (VSV). The virus yields were determined by a plaque assay. In agreement with the results presented above, treatment of the cells with RO 90–7501 alone did not demonstrate any detectable antiviral effect. However, the virus yields are significantly reduced in cells that received combination treatment than in those treated with poly I:C alone (Fig. 8). Similar results were obtained with encephalomyocardititis virus (EMCV) infection (data not shown).

### RO 90–7501 enhances poly I:C-induced activation of MAPKs

**Figure 9:**
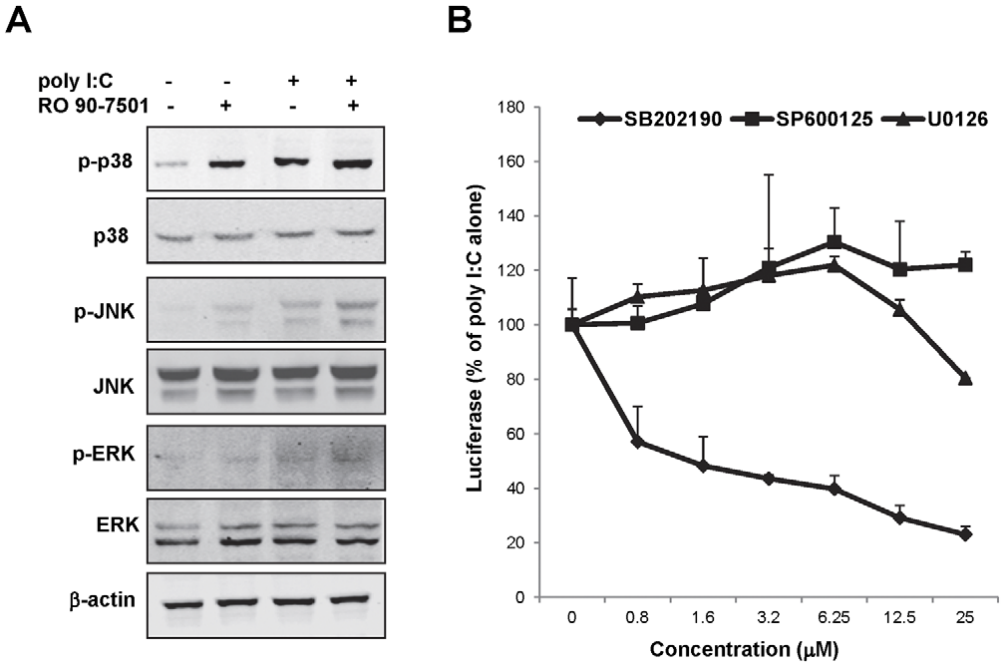
RO 90–7501 enhances poly I:C induced MAPK pathway activation. (A) 293TLR3HA cells were mock treated or treated with 2 µg/ml poly I:C, 10 µM RO 90–7501, alone or in combination for 30 min. A fraction of the cell lysate was analyzed by Western blot for total, phosphorylated p38, ERK and JNK or β-actin on a LI-COR Odyssey system. (B) 293TLR3/IFNβLuc cells were treated with 2 µg/ml of poly I:C in the absence or presence of the indicated concentrations of MAPK pathway inhibitors for 16 h. Luciferase activities were determined and the effects of the tested compounds on the promoter activity were normalized and expressed as a percentage of luciferase activity obtained from the cells treated with poly I:C alone (mean +/− standard derivations, n = 3).

To determine the biological activity of RO 90–7501 on MAPK pathways, 293TLR3HA cells were mock treated, or treated with poly I:C (2 µg/ml), RO 90–7501 (10 µM), alone or in combination, for 30 min. The levels of the total and phosphorylated p38, ERK and JNK were determined by an immunoblot assay. As shown in Fig. 9A, poly I:C treatment was able to activate all three MAPK pathways tested. Interestingly, treatment of the cells with RO 90–7501 alone was able to efficiently activate p38 MAKP and to a lesser extent, ERK and JNK pathways. Furthermore, RO 90–7501 was also able to enhance the activation of the three MAPK by poly I:C. To determine the role of the three MAPKs in poly I:C-induced IFN-β promoter activation, inhibitors of the three MAPKs were applied to poly I:C treated 293TLR3/IFNβluc cells. As shown in Fig. 9B, only the p38 inhibitor (SB202190), but not ERK or JNK inhibitors (U0126 or SP600125), significantly inhibited poly I:C-induced IFN-β promoter activation. This observation, in conjunction with the facts that RO 90–7501 slightly suppresses poly I:C-induced NFκB activation (Figs. 3 and 4) and does not affect poly I:C-induced IRF3 nuclear translocation and activation of IRF3-directed gene (IFIT1 or ISG56) expression (data not shown), implies that activation of p38 MAPK pathway is most likely responsible for the observed enhancement of IFN-β expression and antiviral response by RO 90–7501.

## Discussion

TLRs and RLRs are the principal pattern recognition receptors that sense virus infection and mount a defense by activation of an innate immune response (reviewed in [3],[4]). However, it has also been observed under many circumstances that the TLR- and/or RLR-mediated innate immune response plays a critical role in viral pathogenesis. For example, pneumonia caused by influenza virus or SARS-coronavirus infections [30], [31], [32], hemorrhagic fever caused by dengue virus and many other viruses [2], [33] and encephalitis caused by West Nile virus infection [34] have all been demonstrated to be largely due to the detrimental effects of proinflammatory cytokines induced by the infection itself. Moreover, the identification of TLRs and RLRs as key pathogen recognition receptors for innate immunity has sparked great interest in therapeutic manipulation of the innate immune system. TLR and RLR agonists are being developed for the treatment of cancer, allergies and viral infections, and as adjuvants for vaccines to prevent or treat cancer and infectious diseases [8], [35]. Despite tremendous efforts, only imiquimod (Aldara), a TLR7 agonist, has been approved by US FDA for treatment of papillomavirus-induced genital warts and basal cell carcinoma *via* topical use [36]. Due to the activation of a wide-spectrum of cellular responses, it is not surprising that systematic administration of the TLR agonists in doses necessary to achieve antiviral or anti-tumor effects is usually associated with significant adverse effects [11], [12], [13], [14].

Studies of the signal transduction pathways elicited by TLR and RLR agonists have thus far revealed that induction of type I IFNs and other proinflammatory cytokines is controlled by distinct signal transduction pathways [37], [38]. It is conceivable that pharmacological modulation of TLR and/or RLR signal transduction pathways may alter the cytokine profile induced by the PRR agonists and thus differentially promote the beneficial innate immune response, but limit the detrimental effects. In our effort towards discovery of molecules with such a pharmacological property, we identified RO 90–7501, a compound that was originally reported as an inhibitor of the amyloid-beta peptide (Abeta) assembly *in vitro* [39]. RO 90–7501 selectively enhances TLR3 agonist-induced type I IFN production in both human kidney epithelia and macrophage cell lines (Fig. 7), but does not promote NFκB activation and chemokine production. As expected, treatment of cells with RO 90–7501 significantly enhanced the antiviral activity of poly I:C (Fig. 8).

Molecular pathway analyses revealed that RO 90–7501 slightly suppressed poly I:C-induced NFκB activation, but did not apparently alter the TLR3 agonist-induced IRF3 nuclear translocation and IRF3 activated gene (ISG56) expression (data no shown). Interestingly, we showed that treatment of 293TLR3HA cells with RO 90–7501 was able to rapidly activate p38 MAPK, and to a lesser extent, ERK and JNK. Although the molecular target and signaling pathways leading to the activation of the MAPKs by RO 90–7501 remain to be determined, it is plausible that RO 90–7501 activation of p38 MAPK, which is essential for TLR3 agonist-induced IFN-β gene expression (Fig. 9), may be responsible for its enhancement of TLR3/RLR response. Ironically, RO 90–7501 only enhances TLR3 response in the presence of its agonist, but fails in the condition that the cells were pretreated with the compound for 3 h, followed by treatment with poly I:C in its absence for an additional 9 h (Fig. 5). These results imply that besides p38MAPK, an additional short-lived cellular response induced by RO 90–7501 may be also required for its innate immune enhancement activity.

In corroboration with the work reported herein, Conforti and colleagues recently showed that *in vivo* injection of poly(A:U), a TLR3 agonist, in combination with an immunochemotherapeutic regimen, was able to inhibit TLR3 positive breast tumor growth [40], [41]. Interestingly, TLR3 agonists can elicit the production of a range of chemokines by tumor cells. While CCL5 blockade improved the efficacy of immunochemotherapy, CXCR3 blockade abolished its beneficial effects. Therefore, this work shows that the opposing innate immune responses mediated by chemokines can be pharmacologically modulated to enhance the anticancer efficacy of TLR3 agonists.

In summary, we and others have demonstrated that pharmacological modulation of TLR3 signaling or function of its downstream chemokines and/or cytokines can alter TLR3 agonist-induced innate immune response and enhance its antiviral and anticancer effects. Regardless of whether RO 90–75401 or its derivatives can be developed as a therapeutic agent to enhance the antiviral response of TLR3 agonists, our study supports the concept that pharmacological modulation of TLR and RLR signal transduction pathways is an attractive approach for antiviral drug development.

## Materials and Methods

### Cell Culture and viruses

HEK293 cells were cultured in DMEM supplemented with 10% FBS, 2 mM L-glutamine, 1.5 g/l sodium bicarbonate, 50 U/ml of penicillin and 50 μg/ml of streptomycin. 293TLR3HA, a HEK293 cell-derived stable cell line expressing HA-tagged TLR3, was purchased from Invivogen and cultured in complete DMEM medium containing 30 µg/ml blasticidin. THP-1 cells were a kind gift of Dr. R. Phillips at Immunotope Inc. (originally purchased from ATCC) and cultured in RMPI 1640 medium containing 10% fetal bovine serum and Penicillin/Streptomycin. HEK-Blue IFN-α/β cells (Invivogen), expressing a secreted embryonic alkaline phosphatase (SEAP) under the control of the ISG54 promoter, were cultured in complete DMEM medium containing 100 µg/ml of Zeocin and 30 µg/ml of Blasticidin. Vesicular stomatitis virus was propagated and titrated as described previously [42].

### Compounds

poly I:C was purchased from invivogen. RO 90–7501, chloroquin, SB202190 and U0126 were purchased from Sigma. LY294002 and SP600125 were from Cell Signaling. IKK-2 inhibitor IV was purchased from Santa Cruz Technology.

### Establishment of reporter cell lines

293TLR3HA cells were co-transfected with plasmids expressing firefly luciferase under the control of a human IFN-β promoter (pIFNβ-Luc) [26] or an artificial promoter containing four repetitive consensus NFκB binding sites (Clontech) and pcDNA3 (Invitrogen) at a molar ratio of 10 to 1. Twenty-four hours post transfection, cells were re-seeded at a density of 10^4^ cells per dish of 10-cm in diameter and cultured with medium containing 500 µg/ml of G-418. Individual G-418-resistant cell colonies were picked at day 16 post transfection. The cell colonies demonstrating the highest levels of poly I:C-inducible luciferase expression (measured with Steady-Glo reagent, Promega) were expanded into cell lines and designated as 293TLR3/IFNβLuc or 293TLR3/NFκBLuc, respectively. In order to create similar cell lines for studying cytoplasmic RIG-I-like receptor-meditated innate immune response, parental HEK293 cells that are devoid of TLR3 were co-transfected with plasmids expressing firefly luciferase under the control of a human IFN-β promoter (pIFNβ-Luc) [26] and pcDNA3 at a molar ratio of 10 to 1. A G-418-resistant clone expressing a high level of luciferase in response to lipofectamine-mediated transfection of poly I:C was identified and designated as 293/IFNβLuc.

### High throughput screening

293TLR3/IFNβLuc cells were seeded at a density of 5×10^4^ cells/well in black wall, flat-bottomed, clear 96-well plates (Corning Inc.). The cells were allowed to grow for 24 h before treatment. In the primary screening, the column 1 and 12 of each 96-well plate were mock treated or treated with 2 µg/ml of poly I:C, respectively. Each of the remaining 80 wells were treated with 2 µg/ml of poly I:C and compound from the library of pharmacologically active compounds (LOPAC) (Sigma) at a concentration of 5 µM. A sub-saturating concentration (2 μg/ml) of poly I:C was used to identify both positive and negative modulators of the TLR3 agonist-induced innate immune response. The firefly luciferase activities were measured after 18 h of dsRNA addition by adding 100 μl/well Steady-Glo reagent (Promega), followed by luminometry in a TopCounter (Perkin Elmer). The raw luciferase activity data from each library compound-treated well were normalized as percentage activity relative to cells treated with poly I:C alone. The compounds that reduced or increased luciferase activity by more than 50% were considered as primary “hits”. The hit compounds were subjected to further evaluation of cytotoxicity with MTT assay (Promega) and confirmation of their ability to inhibit or enhance poly I:C activation of IFN-β promoter with the reporter assay described above at an expanded range of concentrations in triplicate.

### Western-blotting assay

293TLR3HA cells were mock treated or treated with 10 µM RO 90–7501, 2 µg/ml poly I:C, alone or in combination for 30 min and lysed with 1x Laemmli buffer. A fraction of cell lysate was separated on sodium dodecyl sulfate-12% polyacrylamine gels and electrophoretically transferred onto PVDF membrane (Bio-Rad). Membranes were blocked with PBS containing 5% nonfat dry milk and probed with antibodies against total, phophorylated p38, ERK and JNK or β-actin (Cell Signal). Bound antibodies were revealed by incubation with IRDye secondary antibodies and imaging with LI-COR Odyssey system (LI-COR Biotechnology).

### RT-PCR analysis of gene expression

293TLR3HA cells were mock treated or treated with 10 µM RO 90–7501, 2 µg/ml poly I:C, alone or in combination. Total RNA was isolated with TRIzol (Invitrogen) and cDNA was synthesized using Superscript III cDNA Synthesis Kit (Invitrogen) and subjected to PCR amplification of IFN-β (forward primer sequence: 5′-GCAGCTGCAGCAGTTCCAGAA-3′; reverse primer sequence: 5′-GCTAGGAGATCTTCAGTTTCG-3′) and β-actin (forward primer sequence: 5′-GCCCTGGCACCCAGCACAATG-3′, reverse primer sequence: 5′-TTAGGTTTTGTCAAGAAAGGGTG-3′ ) using Platinum Taq polymerase (Invitrogen).

### Detection of secreted type-I IFN

THP-1 cells were mock treated or treated with 10 µM RO 90–7501, 2 µg/ml poly I:C, alone or in combination for 6 h and culture media were harvested. HEK-Blue IFN-α/β cells (Invivogen) were seeded at a density of 5×10^4^ cells per well in 96-well plates. One day post seeding, the cells were cultured with conditioned media harvested from the treated THP-1 cells for 20 h. The levels of SEAP in culture media were determined with QUANTI-Blue assay (Invivogen) by following the instruction of manufacturer (Invivogen).

### Detection of IL-8

IL-8 in culture medium of 293TLR3HA cells treated with doses of poly I:C, 10 µM RO 90 7501, alone or in combination, was measured using a human IL-8 ELISA kit from Invitrogen, following the manufacturer's instruction.

### Cell transfection

293/IFNβLuc cells seeded into 96-well plates at a density of 4×10^4^ cells per well. Twenty-four hours post seeding, cells were mock transfected (no RNA) or transfected with 0.2 µg poly I:C per well with lipofectamine 2000 following the manufacturer's direction (Invitrogen) and mock treated or treated with 10 µM of RO 90–7501 for 16 h.

### Antiviral assay

293TLR3HA cells were seeded into 24-well plates at a density of 2×10^5^ cells per well. Twenty-four hours post seeding, cells were either mock treated or treated with indicated concentrations of poly I:C in the absence or presence of 10 µM of RO 90–7501 for 12 h. Cells were then infected with VSV at a MOI of 0.001. Culture media were harvested 16 h post infection and the virus yields were determined by a plaque assay [42].

### Cytotoxicity assay

To determine the cell viability, a MTT based assay was performed (Sigma) as previously described [43]. 293TLR3/IFNβLuc cells were seeded at a density of 5×104 cells per well in 96-well plates. One day post seeding, the cells were mock treated or treated with a serial dilution of RO 90–7501, ranging from 4 μM to 250 µM, for 16 h. The dose-dependent absorbance values at 570 nm, as indicator of cell viability, were then determined and plotted following manufacturer's instruction.
